# Aminoacyl‐tRNA Synthetases: Variant Classification, Functional Assays, and Emerging Therapeutic Strategies

**DOI:** 10.1002/jimd.70184

**Published:** 2026-04-24

**Authors:** M. I. Mendes, D. E. Smith, V. Spek, A. M. Bosch, W. E. Corpeleijn, M. Engelen, S. A. Fuchs, A. Hadchouel, I. U. Heinemann, R. H. Houtkooper, E. M. M. Hoytema van Konijnenburg, S. Kemp, M. Langeveld, C. D. M. Karnebeek, A. Pop, V. M. Siu, N. I. Wolf, G. S. Salomons

**Affiliations:** ^1^ Laboratory Genetic Metabolic Diseases, Department of Laboratory Medicine Amsterdam UMC Location University of Amsterdam Amsterdam the Netherlands; ^2^ Amsterdam Gastroenterology Endocrinology and Metabolism (AGEM) Amsterdam the Netherlands; ^3^ Department of Pediatrics, Division of Metabolic Diseases Amsterdam UMC Location University of Amsterdam, Emma Children's Hospital Amsterdam the Netherlands; ^4^ Amsterdam Cardiovascular Sciences Institute Amsterdam the Netherlands; ^5^ Department of Child Neurology, Emma's Children's Hospital Amsterdam UMC Location Vrije Universiteit Amsterdam the Netherlands; ^6^ Amsterdam Leukodystrophy Center, Amsterdam Neuroscience, Cellular & Molecular Mechanisms Amsterdam the Netherlands; ^7^ Department of Metabolic Diseases, Wilhelmina Children's Hospital University Medical Center Utrecht Utrecht the Netherlands; ^8^ AP‐HP, Hôpital Universitaire Necker‐Enfants Malades, Service de Pneumologie Pédiatrique, Centre de Référence pour les Maladies Respiratoires Rares de l'Enfant, INSERM U1151 INEM Université Paris Cité Paris France; ^9^ Department of Biochemistry, Schulich School of Medicine and Dentistry Western University London Canada; ^10^ Emma Center for Personalized Medicine Amsterdam UMC Amsterdam the Netherlands; ^11^ Department of Endocrinology and Metabolism, Amsterdam University Medical Center Location University of Amsterdam Amsterdam the Netherlands; ^12^ Department of Pediatrics and Human Genetics, Emma Center for Personalized Medicine, Amsterdam Reproduction and Development Amsterdam UMC Location University of Amsterdam Amsterdam the Netherlands; ^13^ United for Metabolic Diseases Amsterdam the Netherlands; ^14^ Division of Medical Genetics, Department of Pediatrics, Schulich School of Medicine and Dentistry Western University London Ontario Canada; ^15^ Child Health Research Institute London Ontario Canada

## Abstract

Aminoacyl‐tRNA synthetases (aaRS) are essential enzymes that charge tRNAs with their corresponding amino acids, playing a critical role in protein synthesis. All 37 nuclear‐encoded *ARS* genes, comprising both cytosolic (*ARS1*) and mitochondrial (*ARS2*) isoforms, have now been linked to human disease. Pathogenic variants in these genes cause a wide range of phenotypes, from dominant peripheral neuropathies to recessive multisystemic disorders. Despite the high number of *ARS* variants identified, functional validation remains difficult, with over 80% of missense variants classified as VUS in public databases. Additionally, the role of non‐canonical aaRS functions in disease remains an area requiring further exploration. Our laboratory developed a high‐throughput LC–MS/MS‐based aminoacylation assay to measure aaRS activity in patient‐derived fibroblasts, aiding in variant classification. This functional approach has contributed to the diagnosis of nearly 200 patients and has uncovered complex variant effects, including thermolabile and splicing‐defective forms. Therapeutically, amino acid supplementation and dietary interventions have shown effect in select cases, while gene therapy is being explored for dominant ARS‐related neuropathies. Amenability to targeted interventions further underlines the need for correct interpretation of genetic variants, which are increasingly recognized as genetic testing is progressively used in the diagnostic work‐up and functional assays. Additionally, natural history studies are essential to improve diagnosis, understand disease mechanisms, and guide and evaluate personalized treatment. This review underscores the critical need for integrated genomic and functional approaches to advance variant interpretation and therapeutic development in the era of NGS.

## 1 From Sequencing to Significance: Interpreting Aminoacyl‐tRNA Synthetase Variants in the Age of Next‐Generation Sequencing

Over the past decade, advances in high‐throughput sequencing technologies, particularly whole exome sequencing (WES) and whole genome sequencing (WGS), have significantly impacted clinical genetic testing and the molecular diagnosis of (childhood) heritable diseases. Clinical application of these broad screening technologies has yielded an enormous number of novel variants, many of which are of uncertain significance (VUS). The majority (> 80%, according to ClinVar [1]) are missense variants, which are particularly difficult to classify. In most cases, variant classification relies on variant frequency in existing databases and *in silico* tools that assess evolutionary conservation and amino acid similarity. Other tools predict the potential effect on mRNA splicing or draw conclusions from existing literature and variant databases. These approaches often yield only probabilistic predictions rather than definitive conclusions, leaving patients and clinicians with uncertain diagnoses. Segregation analysis is useful for autosomal dominant or X linked disorders, but not so much for autosomal recessive disorders, as the number of children per family is often limited.

To improve variant classification, functional evaluation of the associated pathway is needed [2]. Functional testing can include enzyme or transporter assays, determination of transcript and protein expression levels (with qPCR or western blotting), or broader omics analyses such as metabolomics, lipidomics, transcriptomics, and proteomics. In rare cases, functional assays use minigenes to assess splicing effects or overexpression systems, including CRISPR/Cas9‐modified knockout cells, patient‐derived primary cells, or animal models to evaluate variant impact.

In this manuscript, we highlight the critical role of functional characterization through the lens of the recently recognized group of disorders linked to aminoacyl‐tRNA synthetase (aaRS) genes. The urgency of functional testing for variants in these genes is high because the associated clinical phenotypes are diverse and variable, and the variants identified are often novel and of unknown significance. Moreover, early detection and variant interpretation are crucial in the light of emerging therapies which are most effective when initiated prior to the irreversible disease manifestations.

To improve interpretation of *ARS‐*variants, we developed an aminoacylation assay to simultaneously assess the activity of all cytosolic aaRS enzymes directly in patient‐derived fibroblasts [3]. We are currently optimizing this approach for mtaaRS. This assay measures the activities of all 20 aminoacyl‐tRNA synthetases in a single reaction, enabling assaying multiple patients with defects in different aaRS simultaneously. Additionally, the non‐affected aaRS serve as an internal control of cell quality.

## 2 Aminoacyl tRNA Synthetases

### 2.1 Canonical Function: Role in Protein Synthesis

**FIGURE 1:**
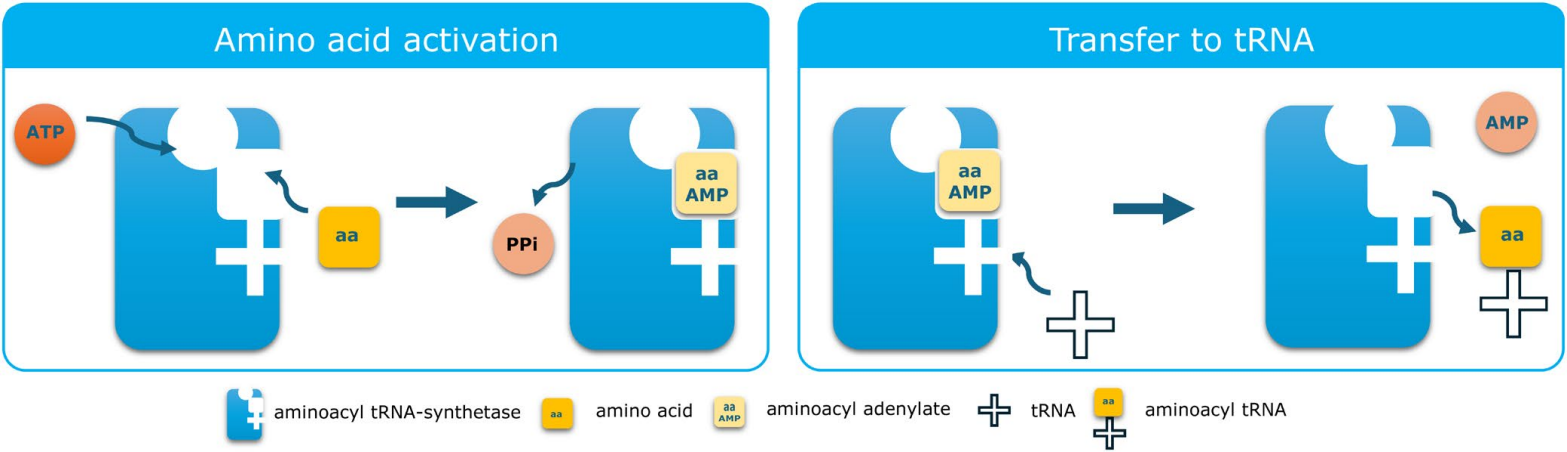
Reaction scheme for the aminoacylation reaction. The first step is the activation of the amino acid with the formation of the aminoacyl adenylate and release of inorganic pyrophosphate. In the second step, the amino acid moiety is transferred to the tRNA to form the aminoacyl‐tRNA.

AaRS are ancient, highly conserved enzymes found in all domains of life. Their canonical function is to catalyze the attachment of specific amino acids to their corresponding tRNAs, a two‐step reaction vital for accurate translation of mRNA into protein (Figure 1). First, the amino acid is activated with ATP to form aminoacyl‐AMP, releasing pyrophosphate. Then, the amino acid is transferred to either the 2′ or 3′ ribose hydroxyl group of the terminal adenine of the tRNA, forming aminoacyl‐tRNA (aa‐tRNA), which is then used during translation [4].

Each of the 20 amino acids included in proteins is typically recognized, activated and charged to its cognate tRNA by a dedicated aaRS. tRNA aminoacylation is catalyzed by different aaRS in the cytosol (from here on referred to as aaRS) and mitochondria (mtaaRS), in total encoded by 37 nuclear genes. Genes are named according to the single letter code of the amino acid followed by “*ARS1*” or “*ARS2*” for aaRS or mtaaRS respectively (e.g., for methionine: *MARS1* and *MARS2*). Protein names use the three‐letter code followed by “RS” for cytosolic proteins (MetRS). Mitochondrial proteins are identified by an additional “mt‐” (mtMetRS). For the sake of simplicity, *ARS* genes and ARS disease will be used in this review to refer to both cytosolic and mitochondrial counterparts.

There are some exceptions to this rule. PheRS is a tetramer, formed by two heterodimers encoded by *FARSA* and *FARSB* genes. The gene *EPRS1* encodes the bifunctional protein responsible for the aminoacylation of both glutamic acid and proline (GluProRS). Regarding the mtaaRS, there is no mtGlnRS. Instead, glutamic acid is attached to tRNA^Gln^ by mtGluRS. Glutamic acid is then converted to glutamine by the glutamyl‐tRNA amidotransferase complex [5]. Lastly, two genes (*GARS1* and *KARS1*) encode both cytosolic and mitochondrial isoforms, with alternative translation start sites that can either include or omit the mitochondrial targeting peptide, determining their final cellular location [6, 7, 8, 9].

Strong structural similarities between certain amino acids can lead to mischarging [10]. Many aaRS have proofreading domains that hydrolyze incorrectly activated amino acids, either before or after tRNA charging [11, 12].

### 2.2 Non‐Canonical Functions

In the cytosol of mammalian cells, ArgRS, GlnRS, MetRS, GluProRS, IleRS, LeuRS, AspRS, and LysRS assemble into the multi‐synthetase complex (MSC), with auxiliary proteins aminoacyl‐tRNA synthetase complex interacting multifunctional proteins (AIMP) AIMP1, AIMP2, and AIMP3 (two units of each protein). AIMPs are important to hold all the components in the complex together through interaction with different synthetases or other AIMPs. Kim et al. [13] established the importance of leucine zipper motifs in the assembly of the complex. Within the complex, in response to specific molecular signals, individual aaRS and AIMPs undergo post‐translational modifications and are released from the MSC to carry out their non‐translational functions [14, 15]. An example is the release of AIMP2 after phosphorylation. Free AIMP2 can move to the nucleus and interact with the tumor suppressor p53, regulating cell death through p53 [16]. Additionally, variants in AIMP2 are associated with progressive neurodevelopmental disorder with microcephaly, seizures, and spastic quadriparesis [17]. The assembly of the MSC appears to be unnecessary for protein translation, suggesting a primary role in supporting or regulating non‐canonical aaRS functions [18, 19]. Gupta and coauthors have recently reviewed this topic [20]. Non‐canonical functions of aaRS are highly diverse and include roles in gene regulation [21], RNA splicing, cell signaling, angiogenesis [22, 23], tumorigenesis [24] and modulation of immune responses [25] and inflammatory responses [26]. These functions are often mediated by domains acquired through evolution [14, 15], while some utilize canonical substrate binding sites. PheRS can catalyze the modification of proteins by aminoacylating lysine residues, as reported for the insulin receptor beta, which is inactivated by the addition of this Phe modification [27]. Some aaRS undergo proteolytic cleavage to perform their non‐canonical functions [28]. One of the most studied examples is the cleavage of TyrRS, the result being a fragment, mini TyrRS, that acts as a proangiogenic cytokine and induces angiogenesis in endothelial cells [29].

## 3 Aminoacyl‐tRNA Synthetase Variants and Human Disease

**FIGURE 2:**
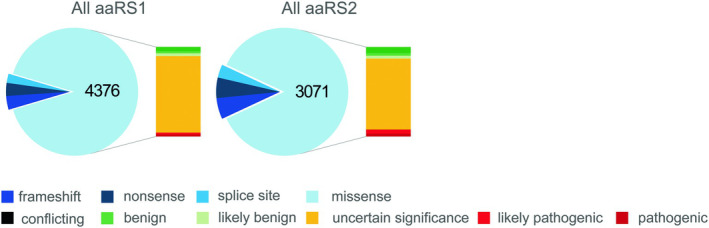
Type of variants in *ARS1* and *ARS2* genes reported in Clin Var (March 2025). As illustrated, most of the reported variants are missense, of which the vast majority is classified as uncertain significance.

With the increasing use of broad sequencing techniques in diagnostics, the number of *ARS* variants is rapidly expanding (Figure 2). As of July 2025, ClinVar reports almost 10 000 *ARS* variants, of which approximately 7500 are missense. Non‐missense variants, including frameshift, nonsense and splice site variants, tend to be easier to classify. However, due to the small changes inherent to missense variants, classification is less straightforward. Among the missense variants reported in *ARS* genes, 84% are classified as VUS and 6% show conflicting interpretation (Figures S1 and S2). Only 2.6% (200) of missense variants are labeled as pathogenic.

Interestingly, variants in *ARS1* genes have been associated with both dominant and recessive disorders. Monoallelic variants in *GARS1* [30], *YARS1* [31], *AARS1* [32], *HARS1* [33], *MARS1* [34], *WARS1* [35], *SARS1* [36] and *NARS1* [37] cause dominant peripheral neuropathies. Biallelic variants in all aaRS and mtaaRS are responsible for recessive disorders, affecting diverse organs or systems. Additionally, dysregulated expression of aaRSs is a characteristic feature in various cancers [38].

### 3.1 Monoallelic (Dominant) Variants

To date, dominant phenotypes are restricted to cytosolic aaRSs that form dimers. Clinically, patients show different types of hereditary motor sensory neuropathy (HMSN), previously denominated Charcot Marie Tooth disease. HMSN has a high incidence in the general population (up to 1:2500). The most common onset is between the ages of 5 and 15, although some patients experience the first symptoms in adulthood. HMSN has been linked to an increasing number of genes, including *AARS1*, *GARS1*, *HARS1*, *MARS1*, *NARS1*, *SARS1*, *WARS1*, and *YARS1*, and more than 60 HMSN disease‐causing *ARS* alleles have been described [31, 32, 34, 39, 40, 41, 42]. The first *ARS1* gene identified as a cause of HMSN was *GARS1*, and this remains the most prevalent. The mechanism by which aaRS variants cause HMSN differs, and both loss of function caused by dominant negative variants and gain of function mechanisms have been described [36, 43, 44, 45]. Vester et al. investigated the function of a dominant variant (p.Arg137Gln) in *HARS1* identified in a patient diagnosed with peripheral neuropathy. Using yeast complementation assay, the studied variant could not complement growth, suggesting that this is a pathogenic loss of function variant. Another mechanism for loss of function was described for *GARS1*, where variants impacted the subcellular localization. There are several possible explanations of how a loss of function variant could cause dominant disease. The variant could interfere with the wild‐type allele, as shown for the dominant negative *NARS1* variant p.Arg543*. Vallee et al. showed that this variant strongly associates with the wild‐type allele, resulting in a predominance of the heterodimer in cells accompanied by a decrease in aminoacylation activity below 50% of controls [46].

### 3.2 Biallelic (Recessive) Variants

Biallelic variants in all 37 *ARS* genes in general cause multisystemic disorders affecting multiple organs. Unlike dominant forms, these are typically associated with early onset and severe disease presentation. Distribution of disease causing missense variants between ancient and modern domains in aaRS showed that the majority were located in the ancient conserved domains [47]. Given their essential role in protein synthesis, one might expect that deficiencies in aaRSs would result in similar clinical presentations across all aaRS deficiencies. To some extent, this holds true: many patients with different aaRS deficiencies exhibit common features such as central nervous system dysfunction, failure to thrive, and feeding or gastrointestinal symptoms [48]. However, certain aaRS deficiencies are associated with more specific phenotypes. For instance, liver disease is frequently observed in patients with deficiencies in IleRS, LeuRS, and MetRS. On the other hand, pulmonary alveolar proteinosis is most often observed in MetRS deficiency. MtaaRS deficiencies present overwhelmingly with neurological symptoms, while symptoms in other organs are less frequent. The reasons why different aaRS deficiencies lead to distinct clinical manifestations remain unresolved. Proposed mechanisms include tissue‐specific differences in codon usage and tRNA isoacceptor abundance, cellular energy constraints in neurons and muscle, and translational demands during development [49, 50], variations in amino acid availability across different tissues [51], and differential aaRS expression levels in specific tissues and cell types [52, 53]. In addition, the extent to which these phenotypes result from disruptions in canonical versus non‐canonical aaRS functions remains unclear. Furthermore, the effects of specific variants on non‐canonical functions may contribute to the complexity of tissue‐specific phenotypes.

Similarly, disease severity and progression between patients with the same aaRS deficiency can be very variable. A clear genotype–phenotype correlation has not been identified for this group of disorders, limiting prognostication.

**FIGURE 3:**
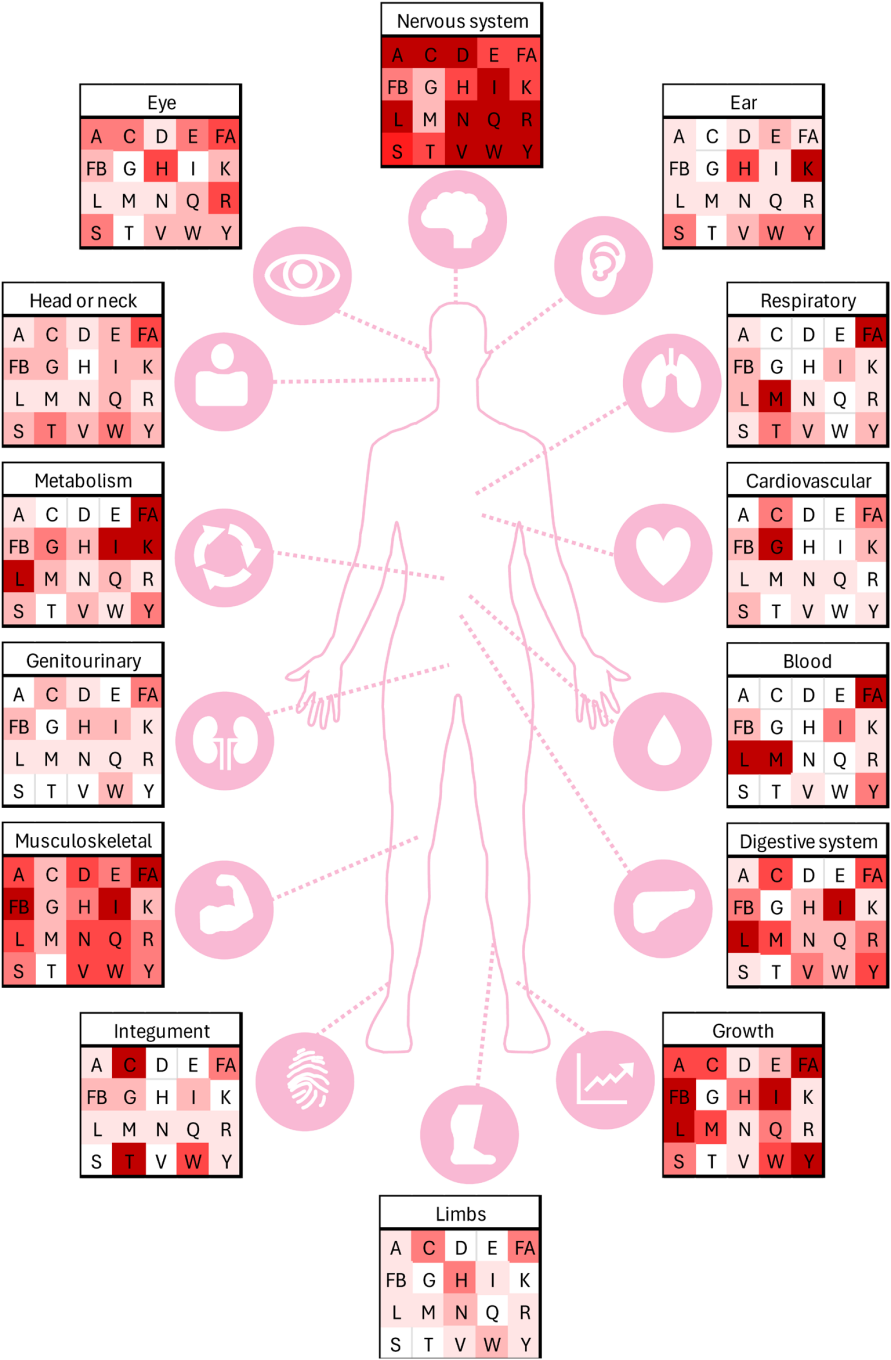
Summary of patient symptoms of 438 aaRS patients described in literature compiled by Hoytema van Konijnenburg et al. [48]. Intensity of the color indicates the percentage of the patients who show these symptoms for the specific aaRS. For a more detailed description refer to the original table by Hoytema van Konijnenburg et al. [48]. Abbreviations: *AARS1* (A), *CARS1* (C), *DARS1* (D), *EPRS* (E), *FARSA* (FA), *FARSB* (FB), *GARS1* (G), *HARS1* (H), *IARS1* (I), *KARS1* (K), *LARS1* (L), *MARS1* (M), *NARS1* (N), *QARS1* (Q), *RARS1* (R), *SARS1* (S), *TARS1* (T), *VARS1* (V), *WARS1* (W), *YARS1* (Y).

Hoytema van Konijnenburg et al. [48] recently thoroughly reviewed 438 aaRS patients described in literature and compiled the observed data in a comprehensive table. Here we represent a summary of that data in Figure 3. They observed that the nervous system is the most frequently affected organ across all aaRS deficiencies (87% of patients). Neurodevelopmental abnormalities (79%), microcephaly (50%), and seizures (46%) are very common. The most described aaRS deficiency is LysRS deficiency (81 patients), while only two patients with variants in ThrRS have been described. Some distinct symptoms are reported in some aaRS deficiencies, such as encephalopathy for AlaRS, LeuRS, and ValRS; neurodevelopmental regression (AlaRS, AspRS, GluProRS, and LysRS); brain hypomyelination (AspRS, ArgRS, and GluProRS); intrauterine growth retardation (CysRS, IleRS, and LeuRS); premature birth (LeuRS); respiratory symptoms (PheRS, MetRS, TyrRS, IleRS, and LeuRS); liver disease (CysRS, PheRS, IleRS, LeuRS, MetRS, and TyrRS); cardiovascular symptoms (GlyRS); anemia (PheRS, LeuRS, TyrRS, IleRS, and MetRS); hypoalbuminemia (PheRS, IleRS, LeuRS, TyrRS, and MetRS); dysmorphias (PheRS and TyrRS); brittle hair (CysRS and ThrRS); and immune system involvement (PheRS and IleRS). For some patients, clinical worsening was triggered by infections (GluProRS, HisRS, LeuRS, and SerRS).

Clinically, recessive mtaaRS variants present symptoms typically associated with mitochondrial dysfunction, including encephalopathies, leukoencephalopathies (some of them with specific MRI patterns allowing diagnosis (mtAspRS, mtGluRS, and mtAlaRS)), sensorineural hearing loss (mtLeuRS), and cardiovascular disease (mtAlaRS). However, certain mtaaRS deficiencies are associated with specific phenotypes as well, such as anemia in mtTyrRS, ovarian failure in mtLeuRS, and kidney disease in mtSerRS.

## 4 Functional Interpretation

### 4.1 Variant Classification Methods

In clinical genetics, *in silico* tools are often the first line of analysis used to assist in variant interpretation. Programs such as PolyPhen [54] and SIFT [55] rapidly classify variants based on evolutionary conservation and the chemical and physical properties of amino acid residues. Splicing prediction tools can assess whether a variant is likely to create or disrupt splice sites. Additionally, whole‐exome sequencing databases, like the gnomAD database [56], provide population‐level data on variant frequency, offering further context for interpretation. More recently, deep learning–based models such as AlphaMissense [57] have been developed to predict the pathogenicity of missense variants with improved accuracy by leveraging large‐scale protein structure and sequence data. These bioinformatic tools are widely accessible and generally free of charge. However, it is important to recognize that all these tools remain predictive in nature and their predictions are not always concordant nor fully accurate; thus, they should be confirmed by family history (segregation analysis) and clinical, biochemical and population data. Although having clear benefits, in silico tools fall short when it comes to mild or complex variants, which is generally the case for patients with variants in *ARS* genes.

### 4.2 Yeast Complementation Assays

*Saccharomyces cerevisiae* is a versatile simplified model system to study the complex biological consequences of aaRS related diseases [58] by investigating impaired translation and the effects on the proteome and cellular viability [59]. Important characteristics of this unicellular eukaryotic organism are rapid growth, genetic translatability and a relatively small genome. Basic processes are very conserved between yeast and human, with approximately half of the genes connected to human disease having yeast homologs [60]. In yeast complementation assays, the specific homologous yeast gene is deleted, and the human gene (wild‐type or carrying a variant) is used to complement. The human protein interacts with the yeast components, altering cellular physiology, which may lead to a phenotype, allowing evaluation of functional effects of specific variants. Alternatively, where the amino acid is conserved in yeast, the variant can be introduced into the yeast protein. Yeast is a relevant in vivo model to test for loss‐of‐function effect as each aaRS allele unable to complement deletion of the yeast ortholog is also unable to charge tRNA molecules in vitro aminoacylation assays, as demonstrated by McLaughlin and coworkers for AlaRS [61].

The clear phenotype observed in yeast models can be a powerful diagnostic tool and a straightforward way to study potential treatment. Furthermore, yeast models have proved to be useful to evaluate the biochemical consequences of variants associated with dominant phenotypes in *AARS1*, *NARS1*, *HARS1*, *GARS1*, *MARS1*, *WARS1*, *YARS1*, reviewed in [58]. More recently, humanized yeast models have been used to understand the underlying molecular mechanism of pathogenic HisRS alleles, identifying mistranslation or defects in dimer formation as disease‐causing mechanisms [62, 63, 64].

### 4.3 Aminoacylation Analysis in Patient Cells

**FIGURE 4:**
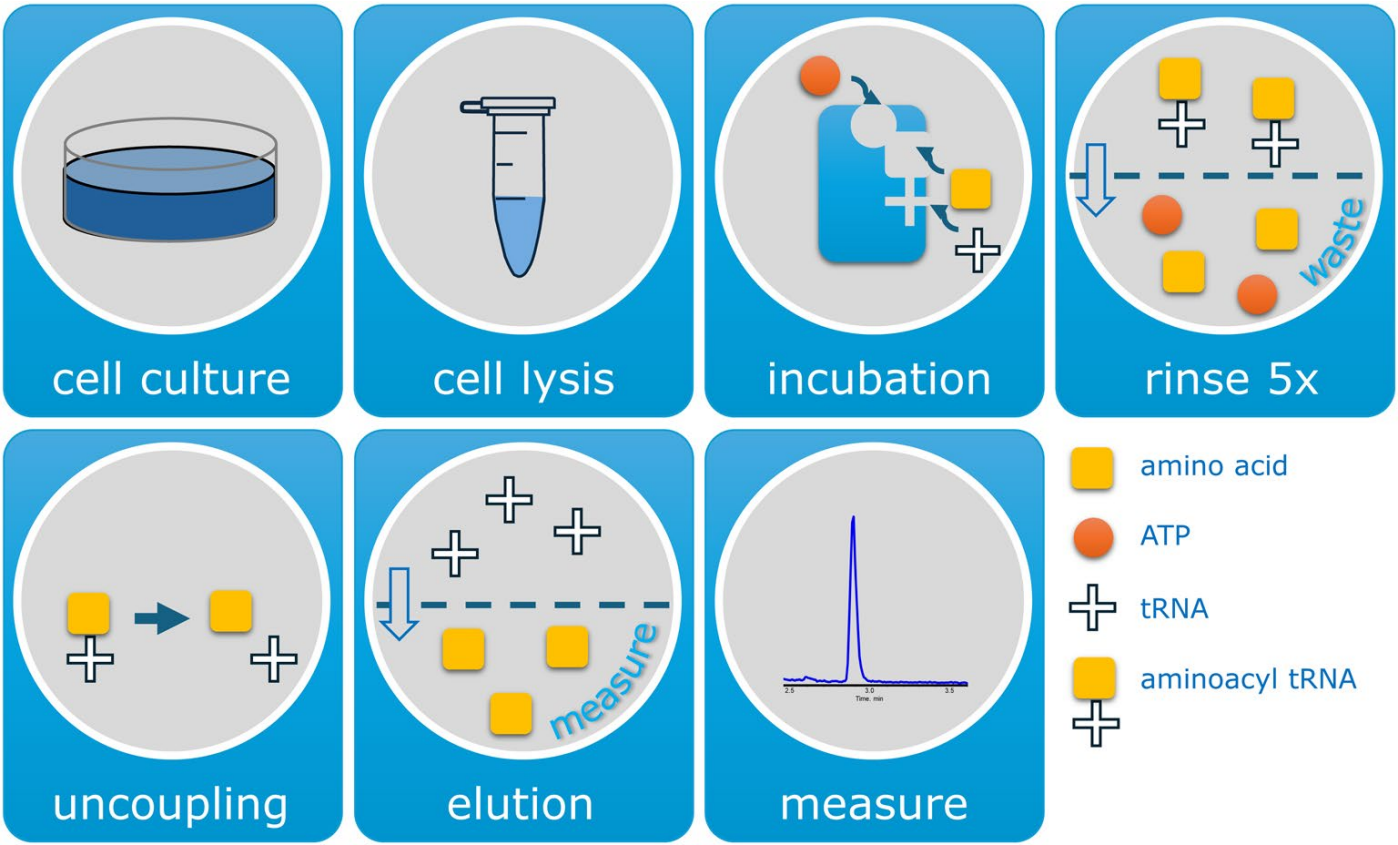
Schematic representation depicting each step of the simultaneous aminoacylation assay. Step 1: Culture of patient derived fibroblasts. Step 2: Lysis of cells by three cycles of freezing–thawing. Step 3: Incubation of the lysate with a mix of the substrates (amino acids, ATP, and tRNA). Step 4: After incubation, the reaction mix is transferred to a 96‐well filter plate filled with TCA in order to stop the reaction (denatured aaRS) and rinsed with TCA to remove unbound amino acids. Step 5: Incubation with ammonia to release amino acids from the tRNA molecules. Step 6: Elution of amino acids with nonafluoropentanoic acid. Step 5: Quantification of amino acids using a Symmetry Shield C18 analytical column (2.0 × 100 mm; 3.5 μm; Waters).

**FIGURE 5:**
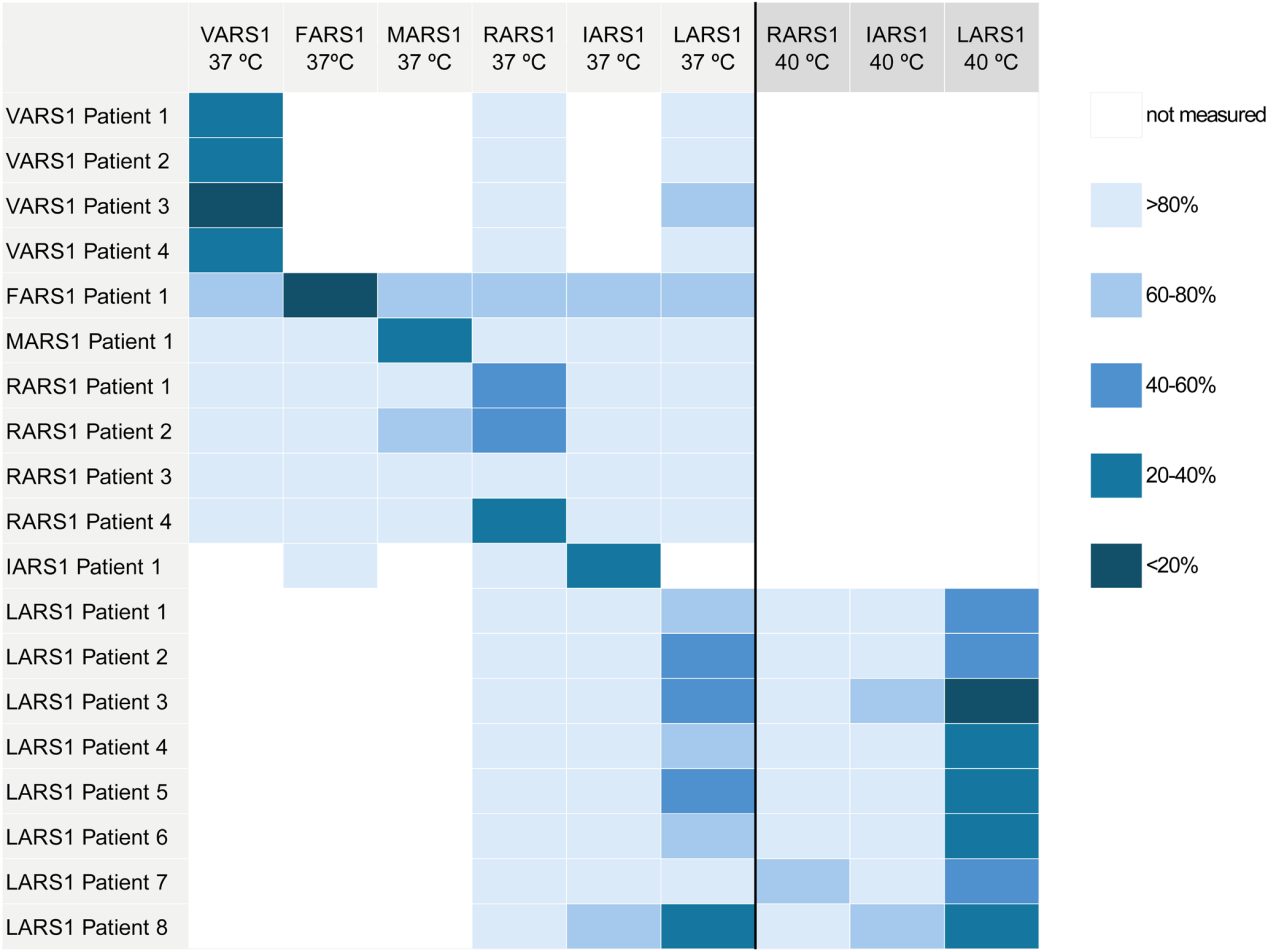
Aminoacylation activities of ValRS, ArgRS, PheRS, IleRS, MetRS and LeuRS for 19 patients with different aaRS deficiencies. Enzyme activities were determined simultaneously in lysates of fibroblasts using an LC–MS/MS method. Results are expressed as a percentage of controls. For LeuRS enzyme activities were also determined following culture at 40°C.

Measurement of aminoacylation activity in patient‐derived cells provides a functional in vitro approach to directly assess the impact of aaRS variants on enzymatic function. In our laboratory, we have developed and implemented the only clinical assay using cells from patients. In this LC–MS/MS assay, the activity of all cytosolic aaRS enzymes is evaluated simultaneously [3]. In short, aminoacylation is assessed by measuring aaRS activity in lysates of fibroblasts from patients. The cytosolic fraction of the lysates is incubated in triplicate for 10 min in a reaction containing ATP, yeast total tRNA, and stable isotopically labeled amino acid mix. The reaction is terminated using trichloroacetic acid (TCA). After washing the samples with TCA, ammonia is added to release the labeled amino acids from the tRNAs. Free amino acids are quantified by LC–MS/MS (Figure 4). This comprehensive assay has proven instrumental in classifying VUS across the full spectrum of *ARS* genes. Since 2018, we have contributed to the diagnosis of nearly 200 patients with ARS1‐related variants, the majority identified through WES. Approximately 25% of these cases have been published. In most instances, the diagnosis is supported by markedly reduced aminoacylation activity, typically less than 30% of control values (Figure 4). However, not all cases present with such clear biochemical deficits. In some patients, residual aminoacylation activity exceeds 30%, complicating variant interpretation and raising the question of how much enzymatic activity loss is required to cause disease. These borderline cases require deeper investigation. For example, in the case of a patient with *RARS1* variants, we identified a near normal activity (RARS1 patient 3 in Figure 5). Further studies revealed the presence of a truncated ArgRS protein, which may function normally in the in vitro assay, but in vivo may have a limited role in protein synthesis. Its function is to add arginine residues to proteins, targeting these proteins to degradation [65]. Similarly, we described a group of eight patients with *LARS1* variants (LARS1 patients 1–8, Figure 5) who showed unexpectedly high LeuRS activity under standard conditions [66]. Clinical deterioration during febrile episodes led us to perform the assay in cells cultured at 40°C, revealing a significant decrease in LeuRS activity, suggesting thermolabile enzyme function and allowing us to classify these *LARS1* variants as temperature‐sensitive pathogenic variants [66]. The identification of thermal sensitive patients led us to include a “stress assay” in our routine diagnostic aminoacylation assay. This consists in the pre‐incubation of the cell lysate at 42°Cprior to performing the assay as described above.

Despite its utility, the LC–MS/MS assay is limited to assessing canonical aminoacylation activity and does not capture potential disruptions in non‐canonical functions of aaRS enzymes. Given the wide spectrum of non‐canonical activities performed by different aminoacyl‐tRNA synthetases (aaRSs), encompassing processes such as transcriptional regulation, signal transduction, and immune modulation, the establishment of a unified assay to evaluate the consequences of variants on these functions is currently not feasible. Consequently, for patients with residual activity exceeding 30%, additional investigations on a case‐to‐case basis are required, and pathogenicity cannot always be definitively confirmed or ruled out.

When material from patients is not available for diagnosis, the use of the yeast complementation assay can be helpful to interpret missense variants of uncertain significance. Specific to mtaaRS, the use of northern blotting and western blotting has been successfully applied to evaluate loss of function missense variants, as reported by Sommerville et al. for the study of mtAlaRS [67].

## 5 Therapy

Current therapies for aaRS deficiencies are largely supportive and aimed at alleviating the symptoms. Among targeted therapies, supplementation with the cognate amino acid and high‐protein diets have been the most extensively explored in patients with aaRS deficiencies [68]. In the following section, we review cognate amino acid supplementation strategies, organized by cytosolic versus mitochondrial aaRS deficiencies and ordered by the number of patients reported in each study.

### 5.1 Cognate Amino Acid Supplementation for Cytosolic aaRS Deficiencies

An overview of published therapy studies, including cognate amino acid supplementation and ketogenic diet interventions in aaRS and mt‐aaRS deficiencies, is provided in Table 1. Cognate amino acid supplementation in 21 patients with cytosolic *aaRS* deficiencies has recently been reviewed by Hoytema van Konijnenburg et al., demonstrating diverse clinical outcomes [48]. Although some clear beneficial effects were reported for most patients (improvements in growth, development, liver and lung disease), amino acid supplementation was insufficient for the most severely affected patients.

**TABLE 1:** Literature overview of case reports of cognate amino acid supplementation and ketogenic diet interventions in aaRS and mt‐aaRS deficiencies.

	*N*	Variant 1	Variant 2	Treatment	Observed Effect	Publication
GlnRS	1	p.Gln515His	p.Arg463^b^	Ketogenic diet	Improved motor skills	Datta et al. (2017)
					Decreased seizure frequency	
HisRS^b^	14	p.Tyr454Ser	p.Tyr454Ser	His supplementation	Improved hearing, vision and overall health	Manuscript in preparation
IleRS	1	c.1305G>C	c.3377dup	Ile supplementation	Increased oral intake	Kok et al. (2021)
		p.Trp435Cys		35–70 mg/kg/day	Decreased vomiting	
					Improved pulmonary function	
IleRS	1	c.1252C>T	c.3521T>A	Ile supplementation	Decreased susceptibility to infections	Kopajtich et al. (2016)
		p.Arg418^b^	p.Ile1174Asn	200 mg/kg/day	Improved development	
LeuRS	1	c.245A>G	c.1118A>G	Leu supplementation 300 mg/kg/day + protein‐fortified diet 2.5 g/kg/day	Improved liver functioning	Casey et al. (2015)
		p.Lys82Arg	p.Tyr373Cys			
LeuRS	1	c.1503 + 3A>G	c.1292T>A	Leu supplementation	Increased oral intake	Kok et al. (2021)
		p.?	p.Val431Asp	25–100 mg/kg/day	Improved liver function, motor functions, strength and resilience during infection	
LysRS	1	c.1786C>T	c.1051C>T	Ketogenic diet	Improved motor skills	Murofushi et al. (2021)
		p.Leu596Ph	p.Arg351Trp			
LysRS	1	c.1160C>T	c.774A>T	Lys supplementation	Improvement in communication	Bejma et al. (2024)
		p.Ser387Leu	p.Arg258Ser	20–30 mg/kg/day + protein‐fortified diet 2.5 g/kg/day	Improved fine motor skills	
					Improved sleep patterns	
LysRS	1	c.1685G>A	c.1412T>C	Lys supplementation 20–30 mg/kg/day + protein‐fortified diet 2.5 g/kg/day	No improvement	Bejma et al. (2024)
		p.Cys562Tyr	p.Leu471Pro			
MetRS	4	p.Ala393Thr	p.Ser567Leu	Met supplementation 80 mg/kg/day	Improved respiratory function, liver function and growth	Hadchouel et al. (2022)
					Reduced systemic inflammation and accumulation of lipoproteinaceous material	
MetRS	3	Not mentioned	Not mentioned	Met supplementation 62 mg/kg/day	No recurrence of lung fibrosis	Roy et al. (2024)
MetRS	2	c.1792C>T	c.1792C>T	Met supplementation 50–125 mg/kg/day	Increased growth	Lenz et al. (2020)
		p.Arg598Cys	p.Arg598Cys		Improved respiratory function	
					Improved liver function	
					Improved pulmonary function	
MetRS	1	c.920A>G	c.1852C>T	Met supplementation dose not mentioned	Improved respiratory function	Rips et al. (2018)
		p.Tyr307Cys	p.Arg618Cys		Increased growth	
					Improved motor skills	
PheRS^a^ (*FARSB*)	1	c.3G>T	c.1118G>C	Phe supplementation 40–100 mg/kg/day	Increased growth	Kok et al. (2021)
		p.Met1?	p.Gly373Ala		Improved liver function	
SerRS	1	c.638G>T	c.638G>T	Ser supplementation 85.7–97.5 mg/kg/day	Improved development	Kok et al. (2021)
		p.Arg213Leu	p.Arg213Leu		Increased height	
TyrRS	1	c.176 T>C	c.237C>G p.Tyr79^b^	Tyr supplementation 100–200 mg/kg/day	No improvements	Estève et al. (2021)
		p.Ile59Thr				
mtAlaRS		c.1699G>T	c.466C>G	Ala supplementation 300 mg/day	No improvements	Catania et al. (2025)
		p.Glu567Ter	p.Leu156Val			
mtAlaRS		c.514 T>A	c.595C>T, c.2188G>A, p.Arg199Cys, p.Val730Met	Aspartate supplementation 300 mg/day	No improvements	Catania et al. (2025)
		p.Phe172Ile				
mtAspRS	2	c.228‐21delTTinsC	c.550C>A	Asp supplementation 1–2 g/day	No improvements	Catania et al. (2025)
		splicing defect	p.Gln184Lys			
mtAspRS	1	c.29 T>C	exon 16–17 del	Asp supplementation 1–2 g/day	No improvements	Catania et al. (2025)
		p.Leu10Pro	mRNA decay			
mtAspRS	1	c.228‐16C>A	c.295‐2A>G	Asp supplementation 1–2 g/day	No improvements	Catania et al. (2025)
		splicing defect	splicing defect			
mtPheRS	1	c.422G>A	c.461C>T	Phe supplementation 450 mg/day	Improvement gross motor skills, movement control and postural stability	Oswald et al. (2023)
		p.(Gly141Glu)	p.(Ala154Val)			

*Note:* Table is alphabetically ordered, starting with aaRS followed by mt‐aaRS and complements the summary provided by Hoytema van Konijnenburg et al. C. and p. notations of the variants are included when reported in the original articles. Rows highlighted in blue indicate cases where treatment was effective. White rows indicate cases where treatment did not result in clinical improvement.

^a^ Patient deceased.

^b^ ClinicalTrials.gov ID NCT02924935.

Currently, the largest cognate amino acid study is a three‐year clinical trial (ClinicalTrials.gov ID NCT02924935) investigating histidine supplementation (50 mg/kg twice daily) in 14 children with Usher syndrome type IIIB caused by the homozygous p.Tyr454Ser variant in *HARS1* (manuscript in preparation, [69]). The disorder is characterized by progressive postnatal hearing impairment and retinal dystrophy, with affected children often experiencing acute deterioration during febrile, typically viral, infections. Histidine supplementation intervention improved hearing, vision, and overall health, illustrating the potential for secondary prevention of symptoms if treatment is initiated at a presymptomatic stage. This clinical trial is not randomized, blinded, or placebo‐controlled due to the extremely small patient population and the absence of alternative therapies for this rare condition.

Several studies have also evaluated methionine supplementation in MetRS deficiency. A cohort of four patients with pulmonary alveolar proteinosis (PAP) and multi‐organ dysfunction received l‐methionine supplementation (80 mg/kg/day), which improved respiratory function (based on clinical evaluation and chest CT), liver function (clinical examination, ultrasound, and biochemical tests), growth and nutritional status, and systemic inflammation (C‐reactive protein, erythrocyte sedimentation rate, blood neutrophil count, and IgG levels) [70]. In a separate report, three patients with MetRS‐related PAP underwent successful lung transplantation without disease recurrence while receiving methionine supplementation (62 mg/kg/day); in contrast to an earlier, similar, patient who did not receive methionine and died due to disease recurrence [71]. Similarly, two siblings with PAP caused by *MARS1* variants received methionine supplementation (50–125 mg/kg/day), which improved growth, pulmonary function, and neurodevelopmental status [72]. Finally, in a single‐patient report, a patient was treated with L‐methionine (dose not reported), resulting in reduced oxygen dependence, fewer hospitalizations, and steady weight gain [73].

However, cognate amino acid supplementation did not confer clinical benefit in every aaRS deficiency. A case report study by Bejma et al. described three patients with LysRS deficiencies, all showing similar clinical symptoms including microcephaly, developmental delay, feeding difficulties, seizures, cytopenias, and nystagmus [74]. Two out of three patients started a lysine supplementation trial (20 mg/kg/day increased to 30 mg/kg/day) along with a protein‐fortified diet of 2.5 g/kg/day. One of the patients showed improvement in communication and fine motor skills (usage of utensils to feed himself and drinking from open cups). However, the second patient showed no improvement upon the same dietary intervention and died 3 months after therapy initiation.

A study by Kok et al. developed personalized amino acid treatments for patients with deficiencies in IleRS, LeuRS, PheRSB, and SerRS [68]. The IleRS patient, an 8‐year‐old boy, exhibited failure to thrive, global developmental delay, autism spectrum disorder, and interstitial lung disease requiring oxygen therapy, with recurrent intensive care admissions for infection‐related respiratory and circulatory failure. Treatment with 35–70 mg/kg/day (in 3 doses) of isoleucine increased oral intake, decreased vomiting, and improved pulmonary function. After prolonged treatment, periods without oxygen therapy increased, and expressive speech and language skills improved. Similarly, LeuRS, PheRS and SeRS patients were treated with leucine (35–100 mg/kg/day), phenylalanine (40–100 mg/kg/day) and serine (85.7–97.5 mg/kg/day) respectively, showing overall improvements in growth and development as well as better clearance of infections. In another case report, a 14‐month‐old patient with a multi‐system disease caused by a TyrRS deficiency, tyrosine supplementation (100 mg/kg/day increased up to 200 mg/kg/day) did not lead to growth improvements or alleviation of other symptoms [75]. Another study reported on a patient with IleRS deficiency, who appeared less susceptible to infections and showed improved development after isoleucine supplementation (200 mg/kg/day) [76]. Lastly, a patient with LeuRS deficiency manifesting as hypoalbuminemia was given leucine supplementation (300 mg/day) together with a high‐protein diet, yet this approach failed to correct the low albumin levels or other clinical or biochemical parameters [77].

In summary, treatment of ARS1 deficiencies with cognate amino acids shows promise, especially for patients with a somewhat milder phenotype, but it remains challenging to distinguish treatment effects with limited availability of natural history [48]. It is also unclear to what extent neurological symptoms can be improved. More studies are needed to rigorously assess efficacy, ideally with proper control arms or crossover designs. However, the extremely small patient populations and, among other reasons, the reluctance of parents and caretakers to discontinue a therapy, especially when clinical improvement is observed, make such studies particularly challenging in this field.

### 5.2 Cognate Amino Acid Supplementation in Mitochondrial aaRS‐Deficiencies

With respect to mitochondrial aaRS, studies investigating the effectiveness of cognate amino acid supplementation and a protein‐rich diet are more sparse, with a total of 9 case reports. Recently, a two‐year long pilot clinical trial explored oral supplementation with alanine (150 mg twice daily) and aspartate (500 mg twice daily, subsequently increased to 2 g per day after 4 months) in two adult patients with mtAlaRS deficiency and four adult patients with mtAspRS deficiency. All patients who completed the trial showed no improvement of energy levels, white matter damage, or cognitive capabilities, although some positive effects of amino acid supplementations cannot be excluded as clinical stabilization was observed in five patients [78]. Additional reports of cognate amino acid supplementation in mtaaRS deficiencies are limited to individual patients. An N‐of‐1 trial was designed for a 3‐year‐old girl with mtPheRS deficiency, presenting with developmental delay and growth retardation. She received oral phenylalanine (150 mg three times daily). Overall, the dietary supplementation improved gross motor function, mobility, postural control, and overall quality of life [79]. These improvements declined during the treatment break and re‐emerged after treatment was restarted, strongly suggesting a link between amino acid intake and amelioration of the disease.

### 5.3 Alternative Therapy Approaches

Other personalized treatment approaches have also been explored. A ketogenic diet has shown improvements of motor skills and social engagement for patients with GlnRS deficiency [80] and LysRS deficiency [81]. Additionally, supplementing cognate tRNA molecules has been tested in model organisms with positive outcomes [82]. Supporting this concept, overexpression of tRNA^Gly‐GCC^ in CMT2D mouse models improved body weight and motor performance as well as reduced nerve conduction velocity and compound muscle action potential amplitude [83]. Despite current challenges in delivering tRNA‐based therapeutics, this form of therapy could be a potential alternative for patients who do not respond to amino acid supplementation.

### 5.4 Gene Therapy

Beyond dietary and substrate‐based strategies, ongoing research is also examining whether gene therapy might offer future therapeutic possibilities, although evidence is currently restricted to model systems. Notably, several studies have investigated gene therapy in models of dominant metabolic disorders such as HMSN, including forms caused by aaRS variants (reviewed in [84]). A study by Ozes et al. investigated the efficacy of neurotrophin‐3 gene transfer in mouse models carrying severe and mild *Gars1* variants that exhibit a HMSN phenotype. In the severe variant model, treatment led to significant functional and electrophysiological improvements, accompanied by enhanced myelination, improved neuromuscular junction integrity, increased muscle fiber size, and reduced myopathic alterations [85]. In contrast, the *Gars1* variant associated with milder disease showed less pronounced effects. Morelli et al. also investigated gene therapy approaches for HMSN caused by *GARS1* variants. They identified a de novo *GARS1* pathogenic variant in a patient with severe peripheral neuropathy and generated a mouse model harboring the same variant. Allele‐specific RNA interference targeting the variant *Gars1* transcript nearly completely prevented neuropathy in mice treated at birth [85, 86].

## 6 Current Challenges and Future Directions

Overall, aaRS deficiencies remain relatively newly described disorders, with small patient cohorts and limited information on the natural history [48]. Consequently, establishment of the diagnosis is often delayed, frequently after irreversible damage has occurred. Additionally, the extent to which the clinical symptoms can be treated or prevented by amino acid supplementation or increased protein intake appears to be specific to the affected aaRS. Given the low number of case reports on cognate amino acid supplementation in mtaaRS patients, elucidating its efficacy remains challenging and warrants further investigation. While alternative treatment options are being explored, potentially offering therapy to patients unresponsive to specific amino acid therapy, more studies are needed to translate findings from animal models to human clinical practice. Larger scale or cross‐over clinical studies and further exploration of therapeutic effects for other recessive aaRS diseases are important next steps to improve outcomes. Additionally, with the setup of controlled clinical trials or patient groups or alternatively “N is 1” cross‐over trials, observational bias could be minimized.

## 7 Conclusions

Over the past two decades, pathogenic variants in all 37 nuclear‐encoded *ARS* genes have been implicated in human disease. The increasing use of next‐generation sequencing in diagnostics has led to the identification of numerous variants in these genes, including many of unknown significance, which do not afford the patient a diagnosis. Thus, there is a great need for adequate variant classification, especially since experimental disease modifying therapy is available for some of the aaRS deficiencies, with first evidence pointing toward better outcomes with early therapy initiation. To facilitate interpretation of aaRS variants, we have developed a functional assay capable of directly measuring aminoacylation activity of cytosolic aaRS enzymes in primary cells. Next in line to be developed are comparable assays for mtaaRS and systematic investigation of the functional consequences of variants affecting non‐canonical roles of aaRS enzymes. Finally, to gain insight in the disease spectrum, natural disease course and effect of therapies, larger cohort studies are needed and therapeutic interventions need to be evaluated in a systematic manner.

## Funding

The authors have nothing to report.

## Conflicts of Interest

The authors declare no conflicts of interest.

## Supporting information


**Figure S1:** Type of variants in each *ARS1* gene (pie chart) and further subcategorization within the group of missense variants (column).

**Figure S2:** Type of variants in each *ARS2* gene (pie chart) and further subcategorization within the group of missense variants (column).

## Data Availability

Data sharing not applicable to this article as no datasets were generated or analysed during the current study.
